# New pharmacological strategies in rheumatic diseases

**Published:** 2016

**Authors:** RE Schiotis, AD Buzoianu, DF Mureșanu, S Suciu

**Affiliations:** * Department of Pharmacology, Toxicology, and Clinical Pharmacology, “Iuliu Hatieganu” University of Medicine and Pharmacy, Cluj-Napoca, Romania; ** Department of Rheumatology, Clinical Hospital of Infectious Diseases, Cluj-Napoca, Romania; *** Department of Neurosciences, “Iuliu Hatieganu” University of Medicine and Pharmacy, Cluj-Napoca, Romania; **** Department of Physiology “Iuliu Hatieganu” University of Medicine and Pharmacy, Cluj-Napoca, Romania

**Keywords:** abatacept, clinical efficacy, rheumatoid arthritis, rheumatic diseases, safety

## Abstract

Targeting the pathogenic pathway of chronic inflammation represents an unmet challenge for controlling disease activity, preventing functional disability, and maintaining an adequate quality of life in patients with rheumatic diseases. Abatacept, a novel molecule that inhibits co-stimulation signal, induces an inhibitory effect on the T-cells. This will further interfere with the activity of several cell lines, leading to the normalization of the immune response. In the latest years, abatacept has been extensively investigated in studies of rheumatoid arthritis for which it was recently approved as a second line biologic treatment in Romania. This review presents the clinical efficacy of abatacept in several rheumatic diseases and highlights the safety profile of this biological agent.

**Abbreviations**:

ACR = American College of Rheumatology, ADR = Adverse drug reaction, APC = antigen presenting cell, ApS = psoriatic arthritis, CRP = C reactive protein, CTLA-4 = Cytotoxic T-Cell Lymphocyte Antigen-4, DAS = Disease activity score, DMARDs = Disease modifying antirheumatic drugs, EMA = European Medicine Agency, EULAR = European League Against Rheumatism, FDA = Food and Drugs Administration, HBV = Hepatitis B virus, JIA = Juvenile Idiopathic Arthritis, LDA = low disease activity (LDA), MRI = magnetic resonance imaging (MRI), MTX = methotrexate, RA = rheumatoid arthritis, RCT = randomized controlled trial, SS = Sjogren’s syndrome, TCR = T cell receptor

## Abatacept

**Mechanism of action**

**Fig. 1:**
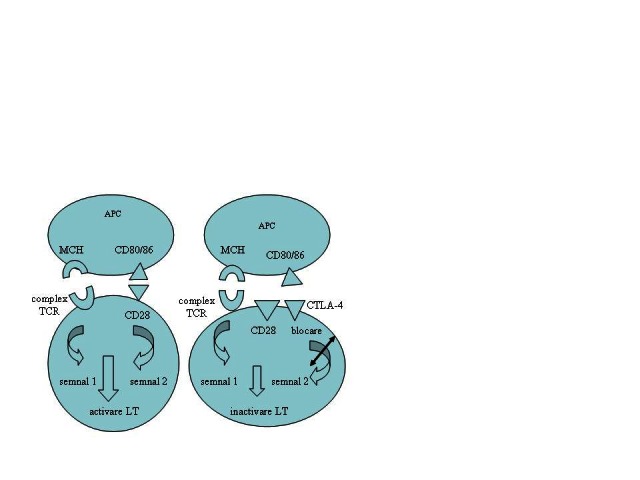
Naïve T-cell activation and inactivation

The pathogenesis of rheumatoid arthritis (RA) includes different cell lines from innate and acquired immunity. The role of immune T-cell in the onset and maintenance of immune response in RA is well known [**1**]. Therefore, the activation of CD4 + T cells generate a waterfall of pro-inflammatory cytokine production and stimulate cell proliferation, processes that cause chronic inflammatory changes and consecutive destruction of the joints [**2**] in RA patients. However, for naïve T lymphocyte to be activated, two signals transmitted from the antigen-presenting cell (APC) are needed. The first signal is generated by the binding of a major histocompatibility complex (MHC) to its receptor on the T lymphocyte (TCR). The second signal, a co-stimulation, is achieved by means of numerous transmembrane receptors on the APCs. One of the most important signals of co-stimulation is achieved by binding of the CD80/ CD86 on APCs with CD28 on T lymphocyte [**3**]. After activation, T-lymphocyte expresses the cytotoxic antigen CTLA-4 (Cytotoxic T-Cell Lymphocyte Antigen-4) on surface, which competitively inhibits CD80/ CD86 to bind to CD28 (**Fig. 1**).

In the early 90s, Linsley et al. synthesized a fusion protein using a human IgG1 and a modified Fc region of CTLA4, which was capable of inhibiting the immune response in vitro. This protein was originally known as the CTLA4-Ig and subsequently was named, abatacept [**4**]. The Fc fragment of abatacept is obtained after several mutations to inactivate it, thereby preventing the antibody- and complement mediated cytotoxicity [**5**].

CTLA4 induces an inhibitory effect on the T-cell, which further interferes with the activity of several cell lines, determining: B-cell inactivity, with consequent decrease in autoantibody formation [**6**], decrease of macrophage activation and reduction of pro-inflammatory cytokines in the synovial joint [**7**]. CTLA4 antigen has an antiresorptive effect by binding directly to the osteoclast precursors, which inhibits their differentiation [**8**]. 

Thus, abatacept is the first therapeutic agent of a new class that selectively modulates a co-stimulatory signal required for the full activation of the T cell, leading to a normalization of the immune response. Abatacept was originally studied in transplant rejection and its initial clinical application was in psoriasis. In the latest years, it has been extensively investigated in studies of RA, which were approved by the Food and Drugs Administration (FDA) in 2005 and European Medicine Agency (EMA) in 2007.

## Clinical efficacy and effectiveness

**Rheumatoid arthritis**

The current indication of abatacept for RA is in combination with MTX and includes patients with moderate or severe disease with inadequate response or intolerance to either synthetic Disease modifying antirheumatic drugs (DMARDs) or at least one anti- TNF- alpha agent. If there is no response to the treatment with abatacept during the first six months, the continuation of treatment should be assessed. 

Clinical efficacy

Abatacept efficacy has been demonstrated in numerous placebo-controlled randomized trials (RTC) conducted on short and long term and its effectiveness has been proven in daily clinical practice by analyzing published evidence from disease registries. The table below illustrates the major clinical trials with abatacept to assess its efficacy and safety (**Table 1**).

**Table 1:** Abatacept efficacy in RTCs

**	*MTX naïve*	**	*Inadequate response to MTX*	**	*Inadequate response to anti-TNF alfa *	**
* Parameter *	*AGREE*	*Phase 2B *	*AIM *	*ATTEST*	*ATTAIN *	*ARRIVE*
**	*1year*	*1year*	*1year*	*6 months*	*6 months*	*6 months*
Studied population	509 RA ABA10mg/ kg+ MTX	339 RA ABA10mg/ kg+ MTX	652 RA ABA10mg/ kg+ MTX	431 RA ABA10mg/ kg+ MTX	393 RA ABA10mg/ kg+ MTX	1046 RA ABA10mg/ kg+ MTX, no WO
Comparator	PBO+MTX	PBO+MTX	PBO+MTX	IFX3mg/ kg+MTX	PBO+MTX	ABA10mg/ kg+ MTX, cu WO
DAS-28 initial, (SE)	CRP 6,3 (1)	CRP 5,5 (0,6)	-	VSH 6,9 (1)	-	CRP 6,2 (0,7)
ACR20/50/70, %	NR/57/43*	63/42/21*	73/48/29*	67/40/21*	50/20/10*	NR
L-DAS, %	54 vs. 37*	50 vs. 22*	43 vs. 10*	21 vs. 11*	17 vs. 3*	22 vs. 23
Remission, %	41 vs. 23*	35 vs. 10*	24 vs. 2*	11 vs. 3*	10 vs. 1*	14 vs. 12
NR = unreported, CRP = C-reactive protein, SE = standard error, Erythrocyte sedimentation rate VSH-, MTX = methotrexate, PBO = placebo, L-DAS = DAS-28 ≤3,2, Remission = DAS-28 ≤ 2.6, IFX = infliximab, WO = washout period, * = statistically significant						

In the abovementioned studies: Phase IIB study [**9**], ATTAIN [**10**], AIM [**11**], ATTEST [**12**], AGREE [**13**], ARRIVE [**14**], abatacept has been proven effective in reducing the signs and symptoms of RA patients who had failed either synthetic DMARDs or TNF-alpha inhibitors, with a maintained efficiency in controlling the disease activity up to 7 years. Abatacept was associated with a reduction in the joint damage and a protective effect against the progression of structural damage over time. Thus, the AIM study found a statistically significant reduction in the rate of radiological progression in patients with active RA refractory to methotrexate (MTX) at 1 year, 45% for erosions and 48% for radiological score (Genant score index), which suggested a slower action of abatacept compared to the anti-TNF alpha agents [**15**]. These results were confirmed in the AGREE trial conducted on early RA patients naïve to MTX, associating negative prognostic factors (anti-CCP antibody and erosions on conventional radiography). The study showed a reduction of about 40% of the total radiological progression, with no radiographic progression after 1 year of treatment with abatacept + MTX in approximately 60% of patients [**14**]. Regarding the effect on long-term structural progression, abatacept was analyzed in an extension of AIM study that showed a significant reduction in the rate of progression at 2 years, far more important in patients who received treatment with abatacept from baseline (1.07 and 2.40 vs. 0.46 in the first year and 0.75 in the second year) [**16**]. Another analysis of patients in the AIM study published in 2011 by Kremer JM et al. showed that total radiological progression of the entire group of patients was significantly lower in the 3rd year of follow up, independent from the initial treatment administered (abatacept and MTX (P = 0.022) [**17**]. 

In the extension of the ATTEST study, it was identified that the rate of progression remains low after 5 years of treatment, but the rates of progression of the group initially on placebo decreased slowly over time, becoming comparable with the group of patients treated from the beginning with abatacept only in the fifth year of the analysis [**18**]. The same effect has been identified in the AGREE study which found that in patients treated with abatacept + MTX, the radiological progression in the 2nd year of analysis was 57%, lower than in the first year. Moreover, this group of patients presented a rate of structural destruction significantly lower in the 2nd year than the patients receiving only MTX treatment (0.84 vs. 1.75 of the total score, P <0.001) [**19**]. 

The ASSET study analyzed abatacept efficacy using magnetic resonance (MRI) as an assessment tool [**20**]. It aimed to assess the effect of abatacept + MTX versus placebo on wrist arthritis on MRI and clinical efficacy and safety of abatacept at 4 months with extension for another 8 months. The treatment with abatacept plus MTX was associated with reduced osteitis, minimal progression of the erosions and the tendency to reduce synovitis compared to placebo + MTX, demonstrating an early effect of abatacept on inflammation and structural damage, with a persistent anti-inflammatory effect and structural protection on MRI [**21**]. Another MRI study carried out by Buch et al. published in 2009, looked at the effect of abatacept on the gene expression of cytokines and cellularity in the inflamed synovium of patients with failure to anti-TNF-alpha treatment. A significant reduction in the DAS28 from baseline (p = 0.01) was registered after 6 doses but the disease activity remained at a high level, suggesting a reduced clinical benefit, however, in accordance with published data for the cohorts resistant to treatment with anti-TNF- alpha [**11**,**22**]. Interesting changes of the synovial joint were identified, namely a reduced expression of pro-inflammatory cytokines (especially IFN-gamma), and reduced tissue perfusion and vascular permeability on MRI, with minimal changes in the structure of inflammatory infiltrate which indicated an anti-inflammatory effect in the synovium, but without altering the local cellular homeostasis.

Head-to-Head trials

So far, there has been only one study that directly compared the efficacy of abatacept with other biological agents. AMPLE is a Phase IIIb, randomized, multinational, investigator-blind, two-year study, whose primary objective was to determine the non-inferiority of abatacept + MTX to adalimumab + MTX in achieving The American College of Rheumatology (ACR20) response at 12 months in patients with moderate and severe RA naïve to biologic treatment [**23**]. The ACR20 response rate was comparable with the 2 biologic therapies at 2 years (59.7% abatacept plus MTX and 60.1% MTX plus adalimumab), with the onset of action of the two agents generally comparable. Radiological progression by analyzing the distribution of the change in the total score from baseline to 2 years showed similar levels of inhibition for both therapies (84.8% in the abatacept plus MTX and 83.8% in the adalimumab plus MTX) [**24**].

Disease registries

 Data on biological treatment of RA patients from Japanese registry were recently published [**25**]. The study analyzed the clinical effectiveness of abatacept (n = 214), adalimumab (n = 175), and tocilizumab (n = 143) in patients with high disease activity (DAS28- CRP> 4.1), moderate and low disease activity for 24 weeks. In patients with a high disease activity at baseline, the clinical efficacy of abatacept was similar to adalimumab and tocilizumab. It was reported that the status of the basal activity in RA significantly influenced the clinical efficacy of abatacept. The authors concluded however, that abatacept could be used to treat patients with RA regardless of the degree of disease activity. Similar effectiveness for abatacept and tocilizumab were reported in RA patients evaluated in daily practice by analyzing the data from the Danish register of biologic, DANBIO [**26**]. Thus, the proportion of patients who obtained a low disease activity (LDA) or remission on week 24 was reduced in patients with a highly active disease at baseline. The basal status of high disease activity has been identified as an independent predictor for not achieving LDA and remission in RA patients included in DANBIO. Similar results have been reported in controlling high disease activity for abatacept, for TNF-alpha blockers and for tocilizumab, the authors suggesting that the clinical effectiveness in controlling the high disease activity is comparable among drugs [**27**-**29**]. The authors concluded that the basal clinical factors are more important in predicting clinical effectiveness in patients with a highly active disease than the biologic treatment administrated.

ACTION is an observational, prospective study, which included European and Canadian RA patients from daily clinical practice, to evaluate the effectiveness of abatacept and persistence of patients on this treatment [**30**]. The results obtained in the control of the disease activity were similar to those reported by Danish DANBIO and French, ORA [**31**] with 67% of the patients achieving good to moderate response to treatment according to EULAR criteria, whether abatacept was first or second line of treatment. Anti-CCP positivity was associated with a better response to abatacept, independently from disease activity [**32**]. Moreover, the clinical response and remission rates were similar to the results obtained in two RCT mentioned above, ATTAIN and ARRIVE, which examined RA patients with inadequate response to anti-TNF-alpha. Persistence rates on abatacept were increased for both first line treatment (93.0%) and second line treatment (> 80.0%), consistent with data from clinical trials [**33**]. The authors suggested that patients treated early with abatacept benefit more from this treatment than patients treated after anti-TNF- alpha failure.

**Juvenile Idiopathic Arthritis (JIA)**

In 2008, FDA approved abatacept alone or in combination with synthetic DMARDS in children aged 6 years with moderate or severe polyarticular JIA. In 2010, EMA approved abatacept in combination with MTX in children with failure to synthetic DMARDs and at least one anti-TNF-alpha. Vaccination with live vaccines should be avoided during and up to 3 months after treatment.

Studies that investigated the efficacy of abatacept in JIA have identified a similar proportion of patients achieving ACR30 Pediatric as with anti-TNF-alpha [**34**-**36**]. Compared with TNF-alpha treatment, the onset of action abatacept was delayed; it took a longer time to relapse and a smaller proportion of patients relapsed after stopping the treatment [**37**]. 

**Early arthritis**

Although the AGREE study identified abatacept effective in the treatment of early arthritis in patients naïve to synthetic DMARDs with severe prognostic factors, there is no indication for the use of abatacept in this clinical situation [**14**]. 

**Psoriatic arthritis**

The role of T lymphocyte in the pathogenesis of psoriasis and psoriatic arthritis (ApS) is well recognized [**38**,**39**]. Activated T lymphocytes are abundantly present in inflamed joints of the patients with ApS and they release the same pro-inflammatory cytokine profile as in patients with RA [**40**]. Phase I of the study, published in 1999 by Abrams et al. has shown promising results in improving skin lesions in patients with refractory psoriasis [**41**]. Less encouraging results have been registered in ApS [**42**]. A phase II of the study published in 2011 by Mease et al. analyzed the effect of abatacept in patients with ApS refractory to synthetic DMARDs and TNF-alpha blockers. The proportion of patients who achieved ACR20 response compared to placebo was greater but the number of patients with statistically significant response was substantially reduced in comparison to the anti -TNF-alpha [**43**-**45**]. However, due to the safety profile of abatacept it may be considered for the management of ApS patients with failure to several biologic DMARDs.

**Axial Spondyloarthritis**

The pilot study that investigated the efficacy and safety of abatacept in patients with active ankylosing spondylitis both naïve and non-responders to anti-TNF alpha obtained disappointing results. Thus, the study did not yield significant improvement in Bath Ankylosing Spondylitis Disease Activity Index (BASDAI) score, the overall disease assessment by the patient, or the value of CRP [**46**]. 

**Lupus nephritis**

In animal studies, abatacept was able to stop the evolution of nephritis. The proposed mechanism was the competitive inhibition of CD28 on the T cell and on the differentiated B lymphocyte (plasma cells) [**47**,**48**]. In the Phase I/ II double-blind, placebo-controlled study conducted in patients with active lupus nephritis receiving background therapy with mycophenolate mofetil and corticosteroids (Bristol-Myers Squibb, IM101075, 2010), the primary endpoint (complete renal response) has not been achieved, and determined the authors to conclude that abatacept is not effective in lupus nephritis. However, the post hoc analysis of the data, suggested there could be a role for abatacept for a particular subset of patients with lupus nephritis, but the low rate of complete response even at that subset indicates a major need to identify drugs that are more effective [**49**]. 

**Sjogren’s Syndrome**

The therapeutic approach to glandular damage in Sjogren’s syndrome (SS) is limited to secretagogue medication [**50**]. For systemic organ damage, the only biological agent that has been successfully used is rituximab. Since CD4 + T cells play an important role in the pathogenesis of SS, abatacept may play a role in the glandular impairment [**51**]. Thus, studies were conducted to investigate the efficiency of abatacept in primary SS in animal and in human models [**52**-**54**]. The newly published study, ROSE, investigated the efficacy and safety of abatacept in patients with secondary SS in patients with RA. It was demonstrated that the treatment with abatacept improved xerostomia, glandular secretory dysfunction, and the antibody production in this group of patients. The early intervention is important in order to regain glandular function. In conjunction with the existing literature data, the authors proposed that abatacept is regarded as a possible new drug, capable of modifying disease progression both in primary SS and SS associated to RA [**55**]. 

## Safety profile

**Adverse effects**

Abatacept has been studied in patients with active RA in placebo-controlled trials that included 1,955 patients with abatacept and 989 with placebo [**56**]. The rate of the adverse drug reactions (ADRs) associated with abatacept in these clinical trials was 7.7 (3.8-13.8) per 100 patients/ year, lower than other biological agents administered intravenously [**57**]. In clinical studies (AIM, ATTAIN, ASSURE [**58**], ATTEST), the percentage of ADRs was roughly similar to placebo (52.2% abatacept vs. 46.1% placebo). The most frequently reported ADRs (≥ 5%) among abatacept treated patients were headache and nausea. The proportion of patients who discontinued treatment due to ADRs was 3.4% for abatacept treated patients and 2.2% for placebo treated patients. Acute infusion-related reactions were mild or moderate and occurred in 0.1% -1% of the patients. These consisted of hypo/ hypertension, dyspnea, nausea, urticaria, flushing, cough, pruritus, rash, wheezing. In the ASSURE study, patients with chronic obstructive pulmonary disease treated with abatacept had respiratory adverse events more frequently compared to the placebo group: 43% vs. 24%. Also, severe respiratory ADRs were more frequent in the group treated with abatacept 10% vs. 0% placebo. In the ATTEST study, the percentage of the overall infections was 60% in the abatacept and 68.5% in the infliximab group. A significant difference in the rate of severe infections, namely 1.9% in the group treated with abatacept versus 8.5% in the infliximab was reported. 

 In the meta-analysis of clinical trials with biological agents conducted by Singh et al. in 2011, the overall risk of severe infections with abatacept was numerically small and statistically significant compared to infliximab, tocilizumab, and certolizumab [**60**]. Pneumonia was one of the most severe ADRs in patients treated with abatacept as seen with other biological agents [**35**]. The rate of occurrence in patient / year was dependent on the patient’s clinical profile, but always higher in patients with previous failure to TNF-alpha blocking agents [**59**,**60**]. The number of malignancies in RA patients treated with abatacept was consistent with the rate observed in other cohorts similar in terms of age and sex distribution. Thus, lung cancer incidence was 0.15 / 100 patient / year, being the most common malignancy and lymphoma incidence of 0.07 / 100 patient / year - the most common hematologic malignancy [**58**]. 

Tuberculosis

The safety of abatacept compared to the anti-TNF alpha agents on the risk of tuberculosis was first demonstrated in an animal model by Bigbee et al. [**61**]. On the other hand, no new case of tuberculosis with abatacept after failure to anti-TNF-alpha was registered in the ARRIVE study [**14**]. Firm conclusions on the risk of tuberculosis should be avoided at this time due to the low number of treated patients.

**Reactivation of hepatitis**

**Hepatitis B**

The role of CTLA4 antigen in the natural history of hepatitis B (HBV) is well documented. Some haplotypes CTLA-4 T (1722C + 49G), which cause the activation of the antiviral T-lymphocyte response were associated with the spontaneous clearance and healing of HBV, while other haplotypes (+ 6230) determined reduced response of T-cell and have been associated with persistent infection [**62**]. In the series of patients reported by Germanidis et al. the reactivation of HBV occurred at intervals of 2 months from stopping abatacept, which might suggest the time needed to restore the T cell immune properties. The authors also noted that HBV reactivation occurred at HBsAg titers above 300 mIU/ ml, consistent with the key role of T cells in the immune control of HBV [**63**]. A recently published retrospective study that analyzed a series of 8 patients with chronic hepatitis B (HBsAg-positive) identified that patients who did not receive prophylactic antiviral therapy concomitant with abatacept had a reactivation of hepatitis, and the combination of abatacept antiviral therapy was able to effectively control the disease activity [**64**]. However, for the antiviral prophylaxis, agents with low risk of resistance (entecavir or tenofovir) should be used as lamivudine resistance is growing [**65**]. The administration of antiviral therapy when the reactivation occurs (HBsAg positive and/ or increased HBV DNA titer) leads to a relief of symptoms of hepatitis [**66**].

**Hepatitis C**

There is little data about the administration of abatacept in patients with chronic hepatitis C. In an article published in 2010, a favorable result was published without the reactivation of hepatitis C [**67**]. This finding supports the recommendation of ACR on the anti- TNF- alpha use (etanercept, infliximab, adalimumab, golimumab, certolizumab) that can be administered in patients with chronic hepatitis C without producing the impairment of the liver function [**68**].

**Immunogenicity**

Studies have shown a reduced rate of immunogenicity of abatacept administered intravenously with minimal impact on the pharmacokinetics, safety, and efficacy of the drug [**69**]. Theoretically, subcutaneous abatacept could generate increased immunogenicity due to potential differences in antigen presentation [**70**]. However, this was not confirmed by the clinical studies, which have shown that the stopping and the subsequent administration of subcutaneous abatacept was well tolerated, with reduced risk of immunogenicity (10% of the patients) [**71**]. In seropositive patients, subcutaneous administration of abatacept generated no side effects and no loss of efficiency or stopping the treatment. These data are in contrast with the data for other biological agents in which immunogenicity determined a discontinuation of the treatment due to adverse reactions or inefficiency [**72**,**73**].

In conclusion, immunogenicity generated by abatacept is reduced, transient, with low titers of autoantibodies, and independent of concomitant use of MTX. In addition, subcutaneous administration of abatacept has similar immunogenicity to intravenous administration and is constant in time. However, the number of patients who synthesized antibodies was too small to make a meaningful assessment of the drug [**74**].

**Disclosures**

None
